# Effects of Different Human Milk Oligosaccharides on Growth of *Bifidobacteria* in Monoculture and Co-culture With *Faecalibacterium prausnitzii*

**DOI:** 10.3389/fmicb.2020.569700

**Published:** 2020-10-30

**Authors:** Lianghui Cheng, Mensiena B. G. Kiewiet, Madelon J. Logtenberg, Andre Groeneveld, Arjen Nauta, Henk A. Schols, Marthe T. C. Walvoort, Hermie J. M. Harmsen, Paul de Vos

**Affiliations:** ^1^Immunoendocrinology, Division of Medical Biology, Department of Pathology and Medical Biology, University Medical Center Groningen, University of Groningen, Groningen, Netherlands; ^2^Laboratory of Food Chemistry, Wageningen University & Research, Wageningen, Netherlands; ^3^FrieslandCampina, Amersfoort, Netherlands; ^4^Stratingh Institute for Chemistry, University of Groningen, Groningen, Netherlands; ^5^Department of Medical Microbiology, University Medical Center Groningen, University of Groningen, Groningen, Netherlands

**Keywords:** human milk oligosaccharides, co-culture, *Bifidobacterium longum* subsp. *infantis*, *Faecalibacterium prausnitzii*, hMO structure-specific

## Abstract

Human milk oligosaccharides (hMOs) are important bioactive components in mother’s milk contributing to infant health by supporting colonization and growth of gut microbes. In particular, *Bifidobacterium* genus is considered to be supported by hMOs. Approximately 200 different hMOs have been discovered and characterized, but only a few abundant hMOs can be produced in sufficient amounts to be applied in infant formula. These hMOs are usually supplied in infant formula as single molecule, and it is unknown which and how individual hMOs support growth of individual gut bacteria. To investigate how individual hMOs influence growth of several relevant intestinal bacteria species, we studied the effects of three hMOs (2′-fucosyllactose, 3-fucosyllactose, and 6′-sialyllactose) and an hMO acid hydrolysate (lacto-N-triose) on three *Bifidobacteria* and one *Faecalibacterium* and introduced a co-culture system of two bacterial strains to study possible cross-feeding in presence and absence of hMOs. We observed that in monoculture, *Bifidobacterium longum* subsp. *infantis* could grow well on all hMOs but in a structure-dependent way. *Faecalibacterium prausnitzii* reached a lower cell density on the hMOs in stationary phase compared to glucose, while *B. longum* subsp. *longum* and *Bifidobacterium adolescentis* were not able to grow on the tested hMOs. In a co-culture of *B. longum* subsp. *infantis* with *F. prausnitzii*, different effects were observed with the different hMOs; 6′-sialyllactose, rather than 2′-fucosyllactose, 3-fucosyllactose, and lacto-N-triose, was able to promote the growth of *B. longum* subsp. *infantis*. Our observations demonstrate that effects of hMOs on the tested gut microbiota are hMO-specific and provide new means to support growth of these specific beneficial microorganisms in the intestine.

## Introduction

It is widely accepted that breastfeeding is the gold standard for infant nutrition, which offers complete nutrition for the newborn. Mother’s milk contains bioactive components that contribute to the healthy development of the newborn (Le Doare et al., 2018). For these reasons, the World Health Organization (WHO) recommends to feed infants for at least 6 months exclusively with breastfeeding (World Health Organization [WHO], 2002; Walker, 2010). However, for a variety of reasons, there are still over 70% of the infants that cannot be exclusively breastfed (Walker, 2010; Heymann et al., 2013). These non-breastfed infants are most often fed with cow-derived infant formula (Coulet et al., 2014; Aly et al., 2018). Up to now, these cow-milk derived infant formula lack human milk oligosaccharides (hMOs), which are one of the most important bioactive components of mother’s milk. These hMOs are unique to humans and provide numerous health-promoting effects (Bode, 2015; Triantis et al., 2018). Until recently, it was not possible to produce hMOs in large amounts for the application in infant formula, but this lately changed. Major advances have been made in large-scale production of hMOs allowing application of a few abundant hMOs in infant formula (Vandenplas et al., 2018).

The beneficial effects of hMOs in human milk are well-established and numerous (Bode, 2015; Cheng et al., 2019, 2020; Kong et al., 2019). One important effect of hMOs is considered to be the support of growth of beneficial gut bacteria (Thomson et al., 2018). The first year of a baby’s life is critical for the establishment of the intestinal microbiome, and hMOs are an important factor in shaping the gut microbiome in the first year of life (Goldsmith et al., 2015). It is, however, still unclear which and how individual hMOs, which are already applied or considered for infant formula, support growth of individual gut bacteria. *Bifidobacterium* is one of the dominant species in the intestine of healthy breastfed infants, and can represent up to 90% of the total microbiome (Moore and Townsend, 2019). hMOs are specifically known to support the growth of *Bifidobacterium* genus (Thomson et al., 2018), e.g., *Bifidobacterium longum* subsp. *infantis*, a strong hMO user that grows well when cultured with hMOs isolated from human milk as the sole carbohydrate source (Underwood et al., 2015). However, whether individual hMOs currently developed for infant formula can also influence the growth of *B. longum* subsp. *infantis* is unknown.

The infant intestine needs, however, fast colonization not only by *Bifidobacterium* genus but also by other species that contribute to making fermentation products such as short-chain fatty acids (SCFAs) that support metabolism and immunity (Conlon and Bird, 2015). Some bacteria ferment hMO and other carbohydrates to produce SCFAs such as acetate, propionate, and butyrate, which are an important energy source for intestinal epithelium, and modulate epithelial integrity (McLeod et al., 2019). A potent SCFA producer is, for example, *Faecalibacterium prausnitzii*, which produces butyrate. This bacterium colonizes the gut during late infancy and is one of the most dominant bacterial species in the large intestine of healthy adults (Laursen et al., 2017). Although its importance for a healthy gut is broadly recognized, it is unknown how *F. prausnitzii* behaves when exposed to hMOs. It is also not known how *F. prausnitzii* is influenced by already present *Bifidobacterium* genus, and whether it benefits from cross-feeding of hMO fermented by *Bifidobacterium* genus.

To gain more insight into how currently applied or proposed hMOs for infant formula influence growth of several relevant intestinal species, we studied the effects of three hMOs 2′-fucosyllactose (2′-FL), 3-fucosyllactose (3-FL), 6′-sialyllactose (6′-SL) and one hMO’s acid hydrolysate lacto-N-triose (LNT2) on *B. longum* subsp. *infantis*, *B. longum* subsp. *longum*, *Bifidobacterium adolescentis*, and *F. prausnitzii*. We first used individual hMOs as the only carbohydrate source for different bacterial strains in monoculture to investigate whether individual hMOs can modulate single-strain growth. Then, *B. longum* subsp. *infantis* and *F. prausnitzii* were brought into co-culture to study the possible interaction between these two bacteria strains. The fermentation products of *B. longum* subsp. *infantis* and *F. prausnitzii* as well as glycosidic degradation of effective hMOs under mono- and co-culture systems were analyzed.

## Results

### Effects on Bacterial Growth of 2′-FL, 3-FL, 6′-SL, and LNT2 Were Bacterial Strain Dependent

**FIGURE 1:**
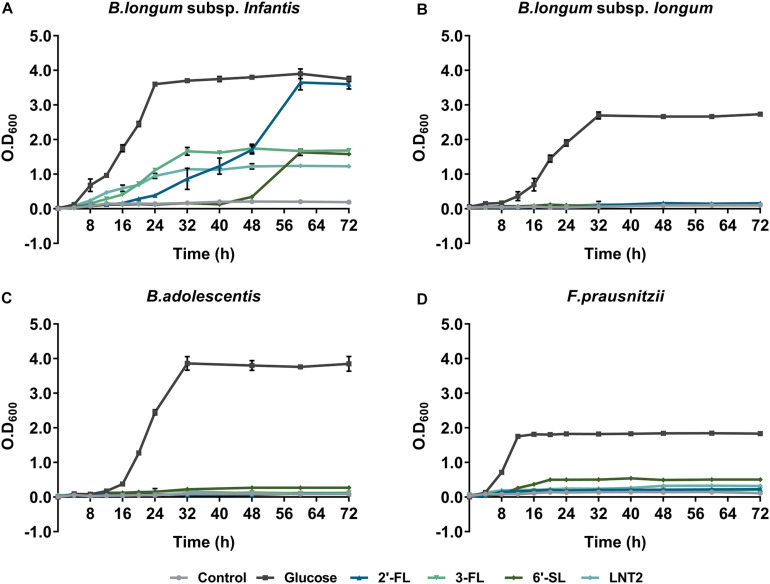
The growth curve of **(A)** *B. longum* subsp. *infantis*, **(B)** *B. longum* subsp. *longum*, **(C)** *B. adolescentis*, and **(D)** *F. prausnitzii* in monoculture, determined by OD_600_. Glucose, 2′-FL, 3-FL, 6′-SL, and LNT2 were included as a sole carbon source as indicated. Basal broth with no carbohydrates added was used as negative control. The assays were carried out three times in duplicate. A representative curve for each condition is shown.

Figure 1 shows the growth curves of *B. longum* subsp. *infantis*, *B. longum* subsp. *longum*, *B. adolescentis*, and *F. prausnitzii* in YCFA broth with glucose, 2′-FL, 3-FL, 6′-SL, or LNT2 as the single carbon source. We found that all bacterial strains were able to grow on glucose, and the effects of 2′-FL, 3-FL, 6′-SL, and LNT2 were variations with the tested strain. *B. longum* subsp. *infantis* could grow on all the substrates we provided, but the effects were hMO structure-dependent (Figure 1A). When grown in the presence of 2′-FL, *B. longum* subsp. *infantis* could reach OD_600_ of 3.6 and reach stationary phase after 56 h of culture. On 3-FL, it grew to an OD_600_ of 1.7 and reached stationary phase after 32 h of culture, which was 24 h earlier than on 2′-FL. With 6′-SL, the growth of *B. longum* subsp. *infantis* started at 48 h of culture, which is much slower than with the other substrates, and reached an OD_600_ of 1.6 after 64 h of culture. On LNT2, *B. longum* subsp. *infantis* grew to an OD_600_ of 1.2 and reached stationary phase after 32 h of culture. In the presence of 2′-FL, the OD_600_ of 3.6 of *B. longum* subsp. *infantis* in stationary phase was significantly higher than with 3-FL, 6′-SL, and LNT2 as the carbon source (*p* < 0.0001, Supplementary Figure S1). In contrast, *B. longum* subsp. *longum* only reached a high cell density (OD_600_ > 1) on glucose, where the OD_600_ was 2.8 after 32 h of culture. None of the tested hMOs was able to support the growth of *B. longum* subsp. *longum* (Figure 1B). The growth behavior of *B. adolescentis* was similar to that observed for *B. longum* subsp. *longum*. On glucose, *B. adolescentis* grew to a high OD_600_ of 3.9 after 32 h of culture, but was not able to grow on the different hMOs and on the hMOs acid hydrolysis product LNT2 (Figure 1C). *F. prausnitzii* reached a lower cell density on the hMOs compared to glucose, and the growth pattern was hMO structure-dependent (Figure 1D). *F. prausnitzii* quickly reached a final cell density at OD_600_ of 1.8 after 12 h of culture on glucose. On 2′-FL and 3-FL, it reached OD_600_ 0.2 in the stationary phase, while with LNT2, it grew to OD_600_ 0.3 at the endpoint. With 6′-SL, the OD_600_ reached 0.5 at the endpoint, which was higher than on 2′-FL, 3-FL, and LNT2, but still significantly lower than on glucose (*p* < 0.0001, Supplementary Figure S1). For the subsequent cross-feeding studies, we selected two bacterial strains to determine whether and which hMOs may impact the growth of the two beneficial bacteria. We chose two essential early life colonizing bacteria; *B. longum* subsp. *infantis*, which is a potent hMO user, and *F. prausnitzii*, which is a major anti-inflammatory commensal bacterium in early life in the gut (Sokol et al., 2008) and a less capable utilizer of hMOs.

### The Co-culture System Can Promote the Growth of the Bacteria

**FIGURE 2:**
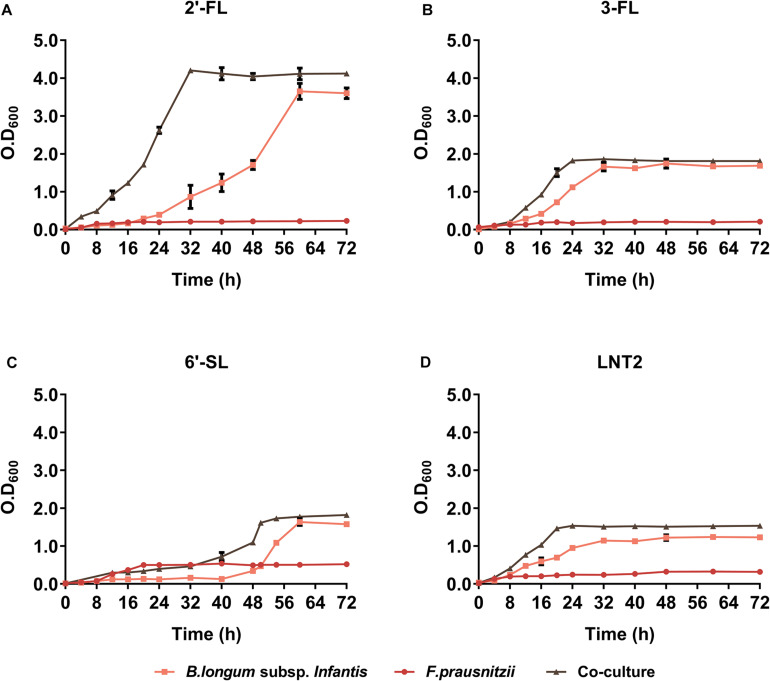
The growth curve of *B. longum* subsp. *infantis* and *F. prausnitzii* in monoculture and co-cultures, determined at OD_600_. **(A)** 2′-FL, **(B)** 3-FL, **(C)** 6′-SL, and **(D)** LNT2 were included as carbon source. The fermentations were carried out three times in duplicate. A representative curve for each condition is shown.

We found that co-culture of *B. longum* subsp. *infantis* and *F. prausnitzii* resulted in different growth rates compared to monoculture and that it was influenced by the type of hMO. In the presence of 2′-FL, co-culture and *B. longum* subsp. *infantis* monoculture cell densities reached similar values (Figure 2A). However, bacteria grew significantly faster in co-cultures than in *B. longum* subsp. *infantis* monocultures, as co-cultures reached stationary phase after 32 h compared to 56 h for the monoculture of *B. longum* subsp. *infantis* (Figure 2A). In the presence of 3-FL, co-culture and *B. longum* subsp. *infantis* monoculture reached a similar cell density, 1.80 and 1.75 at OD_600_, respectively, while the *F. prausnitzii* monoculture reached OD_600_ 0.2 at the endpoint (Figure 2B). Interestingly, in the presence of 6′-SL, the growth curves showed a similar trend as observed with 2′-FL, with the co-culture growing faster than the *B. longum* subsp. *infantis* monoculture, albeit at an overall delayed time. The *B. longum* subsp. *infantis* and *F. prausnitzii* co-culture reached stationary phase after 48 h culturing, while the *B. longum* subsp. *infantis* monoculture just started to grow at this time point. It took 16 additional hours before the *B. longum* subsp. *infantis* monoculture reached the stationary phase. At this faster growth, the OD_600_ in stationary phase was 1.8 in co-culture, 1.6 in *B. longum* subsp. *infantis* monoculture, and 0.5 in *F. prausnitzii* monoculture (Figure 2C). With LNT2 as carbon source, co-cultures and monocultures of *B. longum* subsp. *infantis* reached stationary phases on similar time, but in co-culture, it reached a higher cell density in stationary phase of 1.5 at OD_600_, while in the monocultures of *B. longum* subsp. *infantis* and *F. prausnitzii*, the cell density was only 1.15 and 0.3 at OD_600_, respectively, in the stationary phase (Figure 2D). The different growth rates and final cell density differences in co-cultures and monocultures containing 2′-FL, 3-FL, 6′-SL, and LNT2 as carbon source indicate that the bacteria influence each other’s growth pattern and promote the growth of the bacteria in an hMO structure-dependent way.

### 2′-FL, 3-FL, and LNT2 Do Not Enhance SCFA Production in Co-cultures While 6′-SL Promotes Acetate Production

**FIGURE 3:**
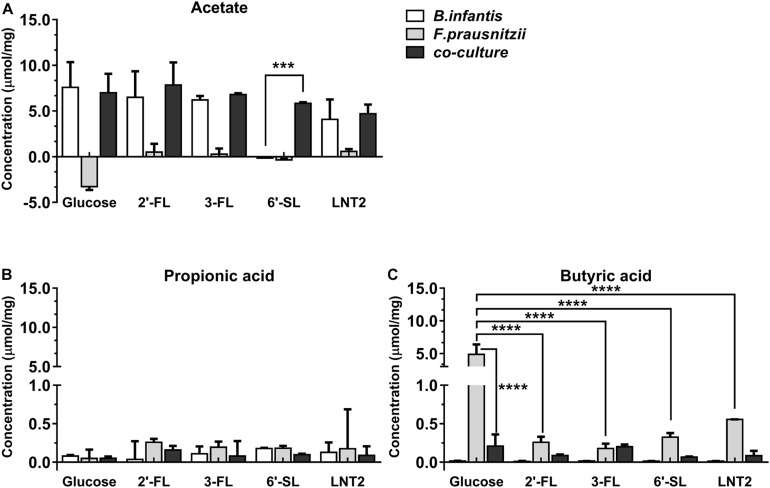
SCFA production of glucose, 2′-FL, 3-FL, 6′-SL, and LNT2 in monoculture and co-cultures of *B. longum* subsp. *infantis* and *F. prausnitzii* after 48-h cultures. The acetate **(A)**, propionic acid **(B)**, and butyrate **(C)** products of *B. longum* subsp. *infantis* and *F. prausnitzii* were measured after 48 h of monoculture and co-cultures when having either 2′-FL, 3-FL, 6′-SL, and LNT2 as carbon source. Glucose served as positive control. Values are changes in concentrations calculated by subtracting the initial values from 0 h. Data are presented as median ± range (*n* = 3). Statistical significance was measured using Kruskal–Wallis test followed by the Dunn’s test and indicated by *p < 0.05, **p < 0.01, ***p < 0.001, or by ****p < 0.0001.

**FIGURE 4:**
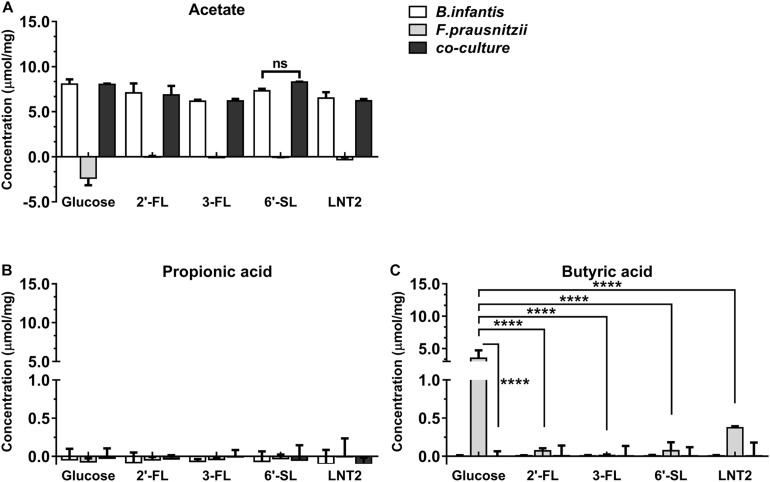
SCFA production of glucose, 2′-FL, 3-FL, 6′-SL, and LNT2 in monoculture and co-cultures of *B. longum* subsp. *infantis* and *F. prausnitzii* after 72-h cultures. The acetate **(A)**, propionic acid **(B)**, and butyrate **(C)** products of *B. longum* subsp. *infantis* and *F. prausnitzii* were measured after 72 h of monoculture and co-cultures when having either 2′-FL, 3-FL, 6′-SL, and LNT2 as carbon source. Glucose served as positive control. Values are changes in concentrations calculated by subtracting the initial values from 0 h. Data are presented as median ± range (*n* = 3). Statistical significance was measured using Kruskal–Wallis test followed by the Dunn’s test and indicated by *p < 0.05, **p < 0.01, ***p < 0.001, or by ****p < 0.0001.

As *B. longum* subsp. *infantis* ferments the hMOs (Ward et al., 2007), we also investigated the production of SCFAs in the mono- and co-cultures (Figures 3, 4). SCFAs are one of the most important metabolic products of the bacteria and reflects activity of the metabolic processes. To this end, we quantified the SCFAs acetate, propionic acid, and butyrate after 48 h and 72 h of monoculture and co-culture. The concentrations of the produced metabolites were calculated by subtracting the initial values at 0 h.

*Bifidobacterium longum* subsp. *infantis* in monoculture with glucose as carbon source (control) produced a high concentration of acetic acid and minor amounts of propionic acid and butyric acid at both 48 h and 72 h. Results were different when 2′-FL, 3-FL, 6′-SL, and LNT2 were used as carbon sources. With 2′-FL, 3-FL, and LNT2 as carbon source in monoculture with *B. longum* subsp. *infantis*, we observed that no significant differences were detected compared to glucose at 48 and 72 h (Figures 3A, 4A). While on 6′-SL, no acetate was detected after 48 h monoculture, and 7.31 μmol/mg acetate was produced after 72 h (Figures 3A, 4A). With *F. prausnitzii*, grown on glucose as carbon source, *F. prausnitzii* consumed acetate in the broth and produced butyric acid and propionic acid in monoculture (Figures 3, 4). With 2′-FL, 3-FL, 6′-SL, and LNT2, only a small amount of butyrate was produced in monoculture. This SCFA production was always significantly lower than on glucose (*p* < 0.0001), but no significant differences between hMOs were observed on both 48- and 72-h cultures (Figures 3C, 4C).

When co-cultures of *B. longum* subsp. *infantis* and *F. prausnitzii* were incubated with the different substrates, high levels of acetate were observed (Figures 3A, 4A). With regard to butyrate production, only a small amount of butyrate was detected with 2′-FL, 3-FL, 6′-SL, or LNT2 after 48 h as well as after 72 h of culture. On glucose, we found similar production rates of acetate when comparing *B. longum* subsp. *infantis* monoculture and co-cultures. However, the butyrate production of the co-culture was significantly lower when compared to *F. prausnitzii* monoculture (*p* < 0.0001) at 48 and 72 h. For acetate production, in the presence of 2′-FL, 3-FL, or LNT2, no differences were found between *B. longum* subsp. *infantis* monocultures and co-cultures. Interestingly, a different result was obtained with 6′-SL. We found that in co-cultures, 5.84 μmol/mg acetate was produced, which was significantly higher than in monoculture of *B. longum* subsp. *infantis* (*p* < 0.0001, Figure 3A), and no acetate was detected in the monoculture of *F. prausnitzii* after 48-h cultures, and the differences between co-culture and monoculture disappeared after 72-h cultures (Figure 4A). With 2′-FL, 3-FL, 6′-SL, and LNT2, only small amounts of butyrate were detected in co-culture, and no significant differences were detected between the groups at 48- and 72-h cultures.

### Co-culture Promotes the Utilization of 6′-SL

As 6′-SL was differently stimulating the production of SCFAs in co-cultures compared to monoculture of *B. longum* subsp. *infantis*, we decided to study and compare the degradation profile of 6′-SL in *B. longum* subsp. *infantis* monocultures and *B. longum* subsp. *infantis* and *F. prausnitzii* co-cultures. To this end, we took samples at 48 and 72 h from the *B. longum* subsp. *infantis* monocultures and *B. longum* subsp. *infantis* and *F. prausnitzii* co-cultures and studied glycosidic degradation in the samples as a measure for carbohydrate utilization.

**FIGURE 5:**
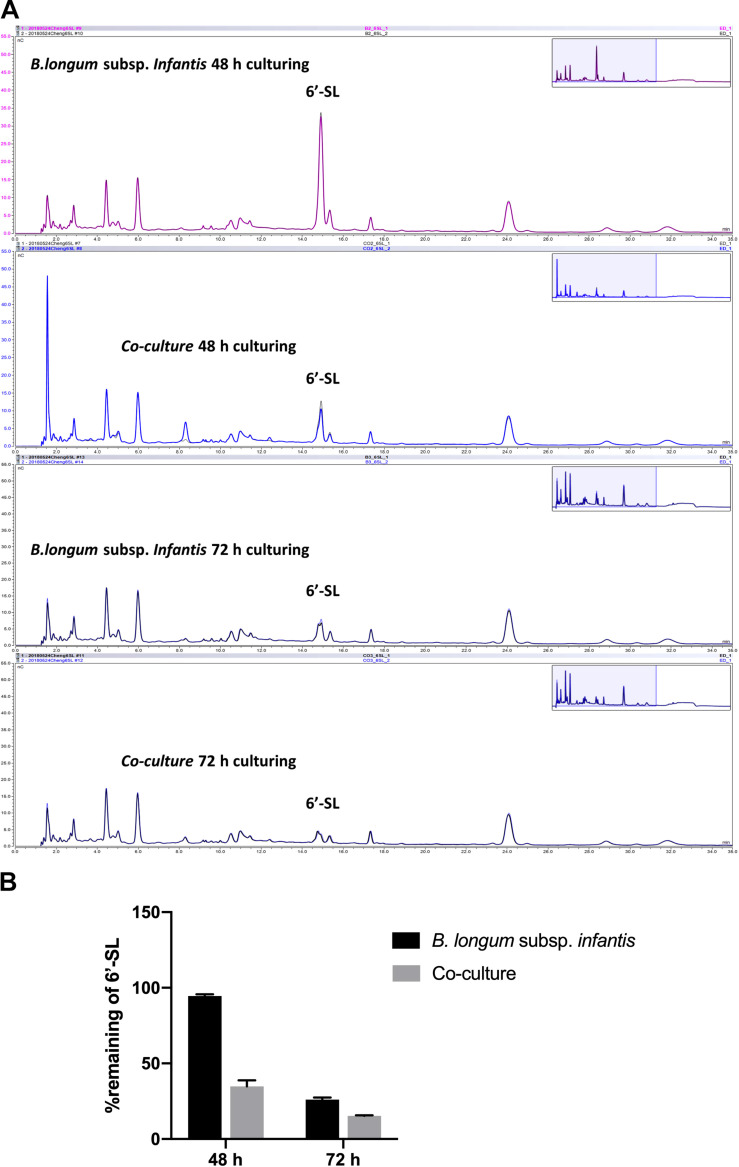
Degradation of 6′-SL after 48-h and 72-h fermentation by *B. longum* subsp. *infantis* and *F. prausnitzii* in mono- and co-cultures. **(A)** HPAEC chromatogram profiles of 6′-SL when *B. longum* subsp. *infantis* in monoculture or co-culture with *F. prausnitzii* after 48 h and 72 h. **(B)** The percentage of remaining 6′-SL in the broth when *B. longum* subsp. *infantis* was in monoculture or co-culture with *F. prausnitzii* after 48 h and 72 h.

As shown in Figure 5, we observed that only small amounts of 6′-SL were utilized in the monocultures of *B. longum* subsp. *infantis* after 48 h, and the peak of 6′-SL was not decreased in high-performance anion exchange chromatography (HPAEC) chromatogram profiles. In contrast, a clear degradation of 6′-SL in the *B. longum* subsp. *infantis* and *F. prausnitzii* co-culture was observed after 48 h (Figure 5A). The quantification results also showed that only 5.4% of the 6′-SL was utilized in the monocultures of *B. longum* subsp. *infantis* after 48 h, while 65.1% of 6′-SL in the *B. longum* subsp. *infantis* and *F. prausnitzii* co-culture was consumed (Figure 5B). After 72 h, 6′-SL 74.0% and 84.7% was used in *B. longum* subsp. *infantis* monoculture and in the co-culture system, respectively.

## Discussion

Human milk oligosaccharides are specifically known to support the growth of beneficial microorganisms in the infant gut (Jost et al., 2015). In particular, *Bifidobacterium* genus (Kirmiz et al., 2018) is acknowledged for that, but which individual hMO and how specific hMOs modulate this process is still unclear. In the present study, our results show that the modulatory effects of individual hMOs on bacterial growth are strongly structure-dependent in both monoculture and co-cultures.

**FIGURE 6:**
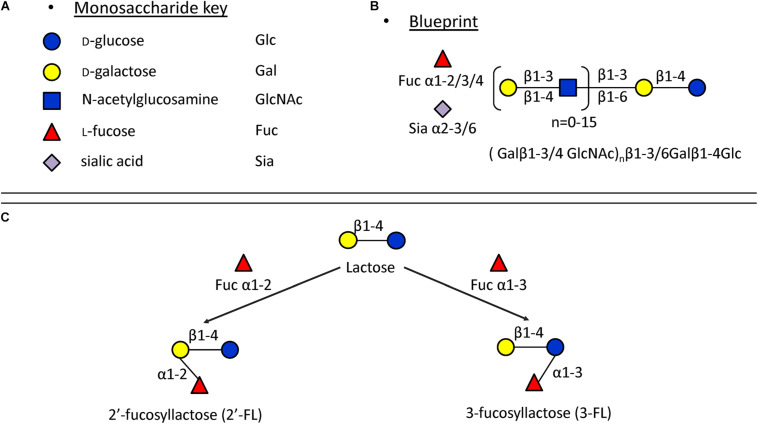
Structures of hMOs. **(A)** Building blocks of hMOs. **(B)** Structural blueprint of hMOs. **(C)** Lactose is fucosylated through different linkages to generate 2′-FL and 3-FL.

In monoculture, the effects of 2′-FL, 3-FL, 6′-SL, and LNT2 on bacterial growth are bacterial strain- as well as hMO structure-dependent. Different growth patterns were observed for different bacteria strains when exposed to the same hMO. The growth of bacteria on 2′-FL, 3-FL, 6′-SL, and LNT2 was hMO structure-dependent. As shown in Figure 6, hMOs are composed of five monomers: D-glucose (Glc), D-galactose (Gal), N-acetylglucosamine (GlcNAc), L-fucose (Fuc), and sialic acid (Sia) (Figure 6A). All hMOs are synthesized from lactose (Galβ1-4Glc), which can be further extended and form more than 200 different structures of hMOs (Figure 6B; Bode, 2015; Thurl et al., 2017). Several studies have demonstrated that the individual hMOs have different effects and that the final outcome of a specific health benefit depends on the composition of individual hMOs (Cheng et al., 2019, 2020; Kong et al., 2019). In the current study, we found that *B. longum* subsp. *infantis* grew faster on 3-FL as carbon source and reached higher cell densities on 2′-FL. 2′-FL and 3-FL are both trisaccharide hMOs that are formed by fucosylation of lactose. The molecules have the same molecular composition and only differ in the attachment position of L-fucose (Fuc) residues on the lactose core region (Figure 6C).

The fermentation of both 2′-FL and 3-FL by bacteria occurs via bacterial α-fucosidase and β-galactosidase enzymes (Thomson et al., 2018), and we observed that the growth rate was higher with 3-FL, and the final cell density was higher with 2′-FL. This indicates different kinetics of fermentation of 2′-FL and 3-FL by *B. longum* subsp. *infantis*. Different enzymes are needed to ferment 6′-SL and LNT2. For fermenting 6′-SL, the bacteria need β-galactosidase and α-sialidase, and fermentation of LNT2 requires β-galactosidase and β-hexosaminidase (Thomson et al., 2018). The different growth patterns of *B. longum* subsp. *infantis* on individual hMOs might be impacted by the catalytic ability of the enzymes. The observation that *F. prausnitzii* reaches a higher final OD_600_ on 6′-SL than when grown with 2′-FL, 3-FL, or LNT2 suggests that *F. prausnitzii* has a higher α-sialidase activity and less α-fucosidase and β-hexosaminidase activity.

*Bifidobacterium longum* subsp. *infantis* produce a high concentration of acetic acid, with minor amounts of propionic acid and butyric acid, while *F. prausnitzii* used acetate in the broth and produced butyric acid (Moens et al., 2016). In the co-culture system, the growth rate was higher than in monoculture of the individual strains. Therefore, we decided to investigate whether the acetate supplied by *B. longum* subsp. *infantis* would stimulate the metabolic activity of *F. prausnitzii* in co-culture. Hence, the SCFA production was quantified and compared between monoculture and co-cultures of *B. longum* subsp. *infantis* and *F. prausnitzii*. Interestingly, under all co-culture conditions tested, no butyrate was detected after 48 h of culture, which suggests that *F. prausnitzii*, while being a potent butyrate producer, was not able to grow in co-culture. This might be associated with the hMO utilization strategy of *B. longum* subsp. *infantis* (Thomson et al., 2018). The utilization of hMOs by *B. longum* subsp. *infantis* is based on the uptake of intact hMOs inside the bacteria, where it is intracellularly degraded (Garrido et al., 2011).

Although *F. prausnitzii* was not able to grow in the co-cultures, we still observed that with 6′-SL as carbon source, *B. longum* subsp. *infantis* and *F. prausnitzii* co-cultures had higher growth rates than in the monocultures (Figure 2C). This indicated that the presence of *F. prausnitzii* may promote the growth of *B. longum* subsp. *infantis* on 6′-SL in co-culture. Since only a small amount of butyrate was produced in *F. prausnitzii* monoculture as well as co-culture (Figures 3, 4), and since there were no statistically significant difference between *F. prausnitzii* monoculture and co-culture, we concluded that 6′-SL was not substantially fermented by *F. prausnitzii*. Therefore the degradation of 6′-SL in *F. prausnitzii* monoculture was not included. This is in accordance with the SCFA production and carbohydrate degradation results, which showed that *B. longum* subsp. *infantis* and *F. prausnitzii* co-culture promotes acetate production and 6′-SL utilization. However, this promoting effect of *F. prausnitzii* was only observed on 6′-SL, and not on 2′-FL, 3-FL, and LNT2. As only the utilization of 6′-SL involves a sialidase (Thomson et al., 2018), we hypothesized that the co-culture of *B. longum* subsp. *infantis* and *F. prausnitzii* might enhance sialidase expression, as only 6′-SL promoted the growth and metabolism of *B. longum* subsp. *infantis*. However, in the current study, only one sialylated hMO was included. Hence, in order to confirm this hypothesis, more sialylated hMOs, such as 3′-sialyllactose (3′-SL), are needed. Unfortunately, due to the technical limitations, pure 3′-SL is not available yet, but would be of great value to test our hypothesis.

In conclusion, we demonstrate that the utilization of individual hMOs as sole carbohydrate sources is bacterial strain- and hMO structure-dependent. Our results show that hMOs currently applied or developed to be applied in infant formula are able to modulate the growth of *B. longum* subsp. *infantis* in a structure-dependent way and stimulate further growth of *B. longum* subsp. *infantis* during co-cultures. In particular, 6′-SL, which can promote the growth of *B. longum* subsp. *infantis* in *B. longum* subsp. *infantis* and *F. prausnitzii* co-culture, showed promising results. Again, we demonstrate that the effects of individual hMOs are highly structure-dependent (Cheng et al., 2019; Kong et al., 2019). Small differences in the molecular structure of hMOs can have significant impact on their biological efficacy. Follow-up studies are needed to identify the specific structure responsible for modulation of bacterial growth, e.g., impact of other sialylated hMOs, which might provide new effective and targeted ways of supporting the growth of beneficial microorganisms in the infant intestine.

## Materials and Methods

### Components

In the present study, 2′-FL (provided by FrieslandCampina Domo, Amersfoort, Netherlands), 3-FL, 6′-SL, and LNT2 (provided by Glycosyn LLC, Woburn, MA, United States) were tested. An overview of the structures of these components are shown in Table 1.

**TABLE 1:** Overview of the structures of selected hMOs.

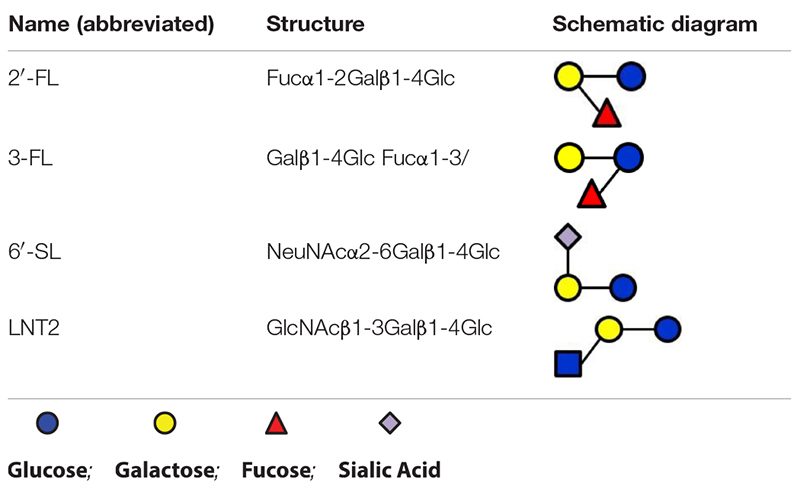

### Bacterial Strains

*Faecalibacterium prausnitzii* A2-165 (DSM 17677) was obtained from the Deutsche Sammlung von Mikroorganismen and Zellkulturen (DSMZ, Göttingen, Germany). *B. longum* subsp. *longum*, *B. longum* subsp. *infantis* ATCC 15697, and *B. adolescentis* (collection strain, NIZO B659) were kindly provided by HH, Department of Medical Microbiology, University Medical Center Groningen (UMCG, Groningen, Netherlands).

### Growth Broth, Monoculture, and Co-culture Conditions

All strains were grown and maintained in yeast extract, casitone, and fatty acid broth (YCFA) (Lopez-Siles et al., 2012). First, the growth and substrate fermentation capacities of the individual strains on different hMO sources were studied. In order to do this, various hMOs were added to the broth at a concentration of 5 mg/ml. YCFA containing 5 mg/ml glucose (Thermo Scientific, Breda, Netherlands) was used as a positive control, while media without carbohydrate served as a negative control. Broth was autoclaved at 121°C for 20 min first, after which the carbohydrate source was added to the cooled broth after sterile filtration. The final pH of the broth was adjusted to 6.5 ± 0.1 by using sterile HCl.

All strains were inoculated in 5 ml of YCFA broth supplemented with glucose as the sole energy source (YCFAG) and incubated anaerobically at 37°C for 24 h. Subsequently, the strains were propagated to 5 ml of YCFAG broth culture overnight as pre-culture; after that, different strains were added to the tubes of YCFA broth supplemented with the different carbon sources at a ratio of 1:100 in duplicate at 37°C. Growth in cultures was monitored spectrophotometrically every 4 h from 0- to 72-h culture by measuring the OD_600_ by using Ultrospec 10 cell density meter (Amersham Biosciences GmbH, Germany).

To study the interaction between *B. longum* subsp. *infantis* and *F. prausnitzii*, co-culture fermentations were performed in YCFA with 5 mg/ml of the different carbon sources. *B. longum* subsp. *infantis* and *F. prausnitzii* cells were inoculated as described above. Subsequently, the strains were propagated to 5 ml of YCFAG media and were pre-cultured separately, and the cell density was monitored spectrophotometrically by measuring the OD_600_ using Ultrospec 10 cell density meter. When OD_600_ reached 1.0 in the pre-culture, *B. longum* subsp. *infantis* and *F. prausnitzii* were added to the same tube of YCFA broth supplemented with the different carbon sources at a ratio of 1:100. Each batch of experiments was made with the same inocula for both *B. longum* subsp. *infantis* and *F. prausnitzii*.

### SCFA Production

Samples for the SCFA analyses were taken at 48 h and 72 h of monoculture and co-culture incubation. The fermentation digest (0.5 ml) was heated at 100°C for 5 min and then centrifuged at 13,200 × *g* for 10 min at room temperature. Analysis of SCFAs (acetate, propionate, and butyrate) by gas chromatography (GC) was done as described previously by Gu et al. (2018). A 250-μl aliquot of the fivefold diluted supernatant of the fermentation product was mixed with 125 μl of a solution containing oxalic acid (0.09 M), HCl (0.3 M), and internal standard 2-ethyl butyric acid (0.45 mg/ml). Afterward, the mixture was allowed to stand at room temperature for 30 min. The temperature profile during GC analysis was as follows: 100°C, maintained for 0.5 min; raised to 180°C at 8°C/min, maintained for 1 min; raised to 200°C at 20°C/min, maintained for 5 min. Xcalibur software (Thermo Scientific, Breda, Netherlands) was used to process data from GC.

### Glycosidic Degradation

Samples for the degradation analysis were taken at 48 and 72 h of *B. longum* subsp. *infantis* monoculture and co-culture. HPAEC was used to measure the carbohydrate degradation as a measure for utilization of carbohydrates by the microorganisms (Cardarelli et al., 2016). An ISC 3000 (Dionex, Sunnyvale, CA, United States), equipped with a 2 × 250 mm Dionex CarboPac PA-1 column and a 2 × 50 mm CarboPac PA-1 guard column, was used for quantification (Albrecht et al., 2009). Briefly, samples were adjusted to a final concentration of 0.05 mg of the substrate per milliliter, and 10 μl of the samples was injected using a Dionex ISC3000 autosampler. The oligosaccharides were eluted (0.3 ml/min) by a gradient of 0–350 mM sodium acetate in 100 mM NaOH for 35 min. Each elution was followed by a washing step with 1 M NaOAc in 100 mM NaOH for 5 min and an equilibration step with 100 mM NaOH for 15 min. A Dionex ED40 detector in pulsed amperometric detection mode was used for detection. 6′-SL at final concentrations of 0.05 mg/ml were used as standards. Chromeleon software Version 6.70 (Dionex) was used for the integration and evaluation of the chromatograms obtained.

### Statistics

The results were analyzed using GraphPad Prism. The normality of distribution of the data was tested by using the Kolmogorov–Smirnov test. Values are expressed as median ± range. Statistical comparisons were performed using the Kruskal–Wallis test followed by the Dunn’s test. *p* < 0.05 was considered as statistically significant (^∗^*p* < 0.05, ^∗∗^*p* < 0.01, ^∗∗∗^*p* < 0.001, ^****^p < 0.0001).

## Data Availability Statement

The raw data supporting the conclusions of this article will be made available by the authors, without undue reservation.

## Author Contributions

LC and PV conceived and designed the experiments. LC and ML performed the experiments and analyzed the data. AG supplied hMOs. LC, MK, ML, AG, AN, and PV participated in the discussion. LC, MK, ML, AG, AN, HS, MW, HH, and PV wrote the manuscript. All authors contributed to the article and approved the submitted version.

## Conflict of Interest

AG and AN are employed by the company Friesl and Campina. The remaining authors declare that the research was conducted in the absence of any commercial or financial relationships that could be construed as a potential conflict of interest.

## Supplementary Material

Supplementary Figure S1 The endpoint OD600 of *B. longum* subsp. infantis, *B. longum* subsp. *longum*, *B. adolescentis* and *F. prausnitzii* in mono-culture. Glucose, 2′-FL, 3-FL, 6′-SL, and LNT2 were included as carbon source. The assays were carried out 3 times in duplicate. Values are expressed as median ± range. Statistical significance was measured using Kruskal-Wallis test followed by the Dunn’s test and indicated by * (*p* < 0.05), ** (*p* < 0.01), *** (*p* < 0.001) or by **** (*p* < 0.0001).
