# Supplementary figures and images for: Effects of Different Human Milk Oligosaccharides on Growth of Bifidobacteria in Monoculture and Co-culture With Faecalibacterium prausnitzii
Source: Front Microbiol. 2020 Oct 30;11:569700. doi: 10.3389/fmicb.2020.569700 (PMC7662573; doi:10.3389/fmicb.2020.569700)

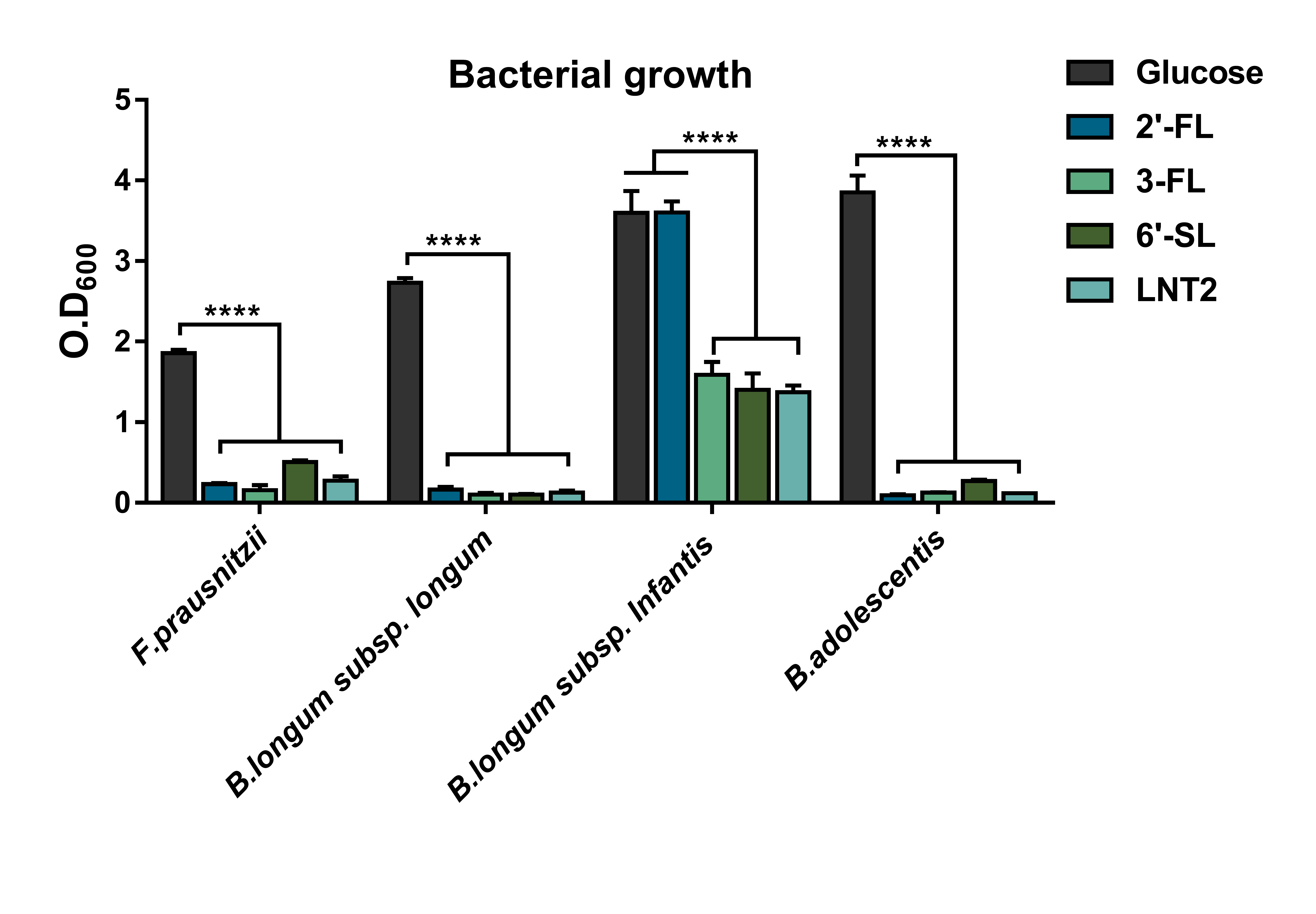

Supplement: Supplementary Figure S1 — The endpoint OD600 of B. longum subsp. infantis, B. longum subsp. longum, B. adolescentis and F. prausnitzii in mono-culture. Glucose, 2′-FL, 3-FL, 6′-SL, and LNT2 were included as carbon source. The assays were carried out 3 times in duplicate. Values are expressed as median ± range. Statistical significance was measured using Kruskal-Wallis test followed by the Dunn’s test and indicated by * (p < 0.05), ** (p < 0.01), *** (p < 0.001) or by **** (p < 0.0001). [file Image_1.tiff]
